# Structural Variation and Chemical Performance—A Study of the Effects of Chemical Structure upon Epoxy Network Chemical Performance

**DOI:** 10.1021/acsapm.1c00378

**Published:** 2021-06-15

**Authors:** Stephen T. Knox, Anthony Wright, Colin Cameron, J. Patrick A. Fairclough

**Affiliations:** † Department of Mechanical Engineering, University of Sheffield, Sheffield S1 4BJ, U.K.; ‡ AkzoNobel, International Paint Ltd, Stoneygate Lane, Gateshead NE10 0JY, U.K.

**Keywords:** epoxy, amine, chemical performance, chemical resistance, ingress, thermoset, chemical structure

## Abstract

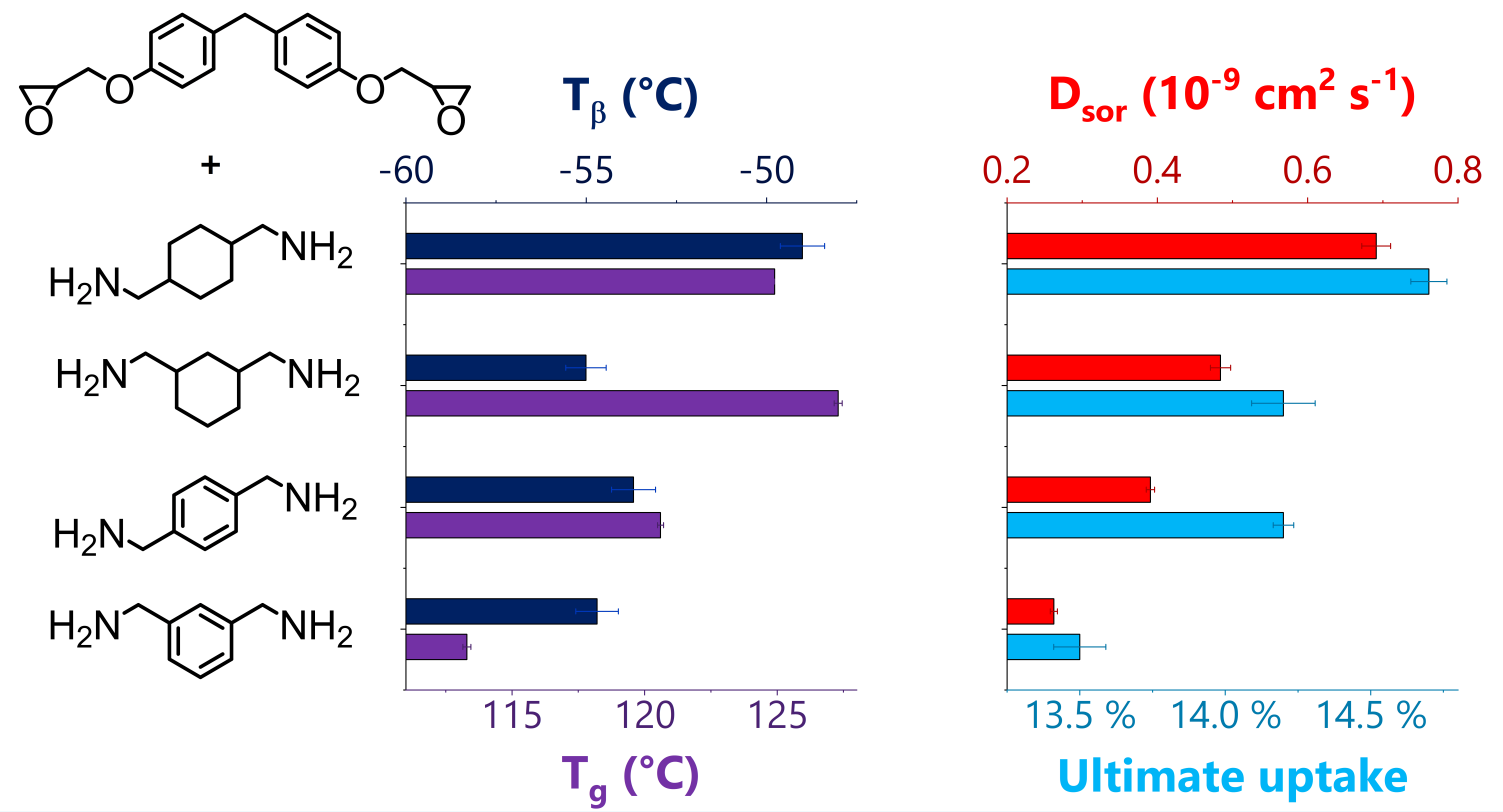

Epoxy resins are used widely as protective coatings, in a wide range of harsh chemical environments. This work explores the influence of subtle structural variation in both epoxy and amine monomers upon chemical performance of cured networks, whether changing molecular geometry, the nature of the chemistry, or the mass between cross-linking reactive groups. To achieve this, four industrially relevant epoxy resins (two based on bisphenol A—Epikote 828 (E828) and Dow Epoxy Resin 332 (DER 332)—and two based on bisphenol F—Dow Epoxy Resin 354 (DER 354) and Araldite PY306 (PY306)) and the isomerically pure para–para-diglycidyl ether of bisphenol F (ppDGEBF) were used to explore variation caused by epoxy monomer variation. Four similar amines (meta-xylylenediamine (MXDA), para-xylylenediamine (PXDA), 1,3-bis(aminomethyl)cyclohexane (1,3-BAC), 1,4-bis(aminomethyl)cyclohexane (1,4-BAC)) were used to explore any variations caused by regioisomerism and aromaticity. Bisphenol F-based resins were found to outperform bisphenol A-based analogues, and chain extension within the epoxy component was found to be detrimental to performance. For amines, 1,3-substitution (vs 1,4) and aromaticity were both found to be beneficial to chemical performance.

## Introduction

Epoxy resins find use in a wide range of applications, including adhesives, protective coatings, and matrixes in fiber composites. Their wide use stems from the robust chemistry that forms highly cross-linked networks. Resistance to chemical attack is a key application of these networks—the ability of a network to withstand this attack is referred to as the chemical performance. Since the cross-linked network produced is insoluble, an important damage mechanism is driven by solvent swelling, which induces a stress in the network which can lead to failure.^1,2^ Gravimetric measurement of solvent sorption and desorption can be used to qualify the chemical performance, in terms of both the rate and total amount of sorption. This is particularly relevant when considering solvent-resistant coatings which may be exposed to a range of chemical aggressors. The uptake of solvent may lead to softening of the coating, which in turn can cause issues such as blistering or breakdown of that lining.

While, in broad terms, it is the formation of a robust high cross-link density covalently bonded network that enables the high chemical performance for epoxy networks, there are a range of factors within that framework which affect the chemical performance. For example, changes in free volume and network polarity have both been shown to influence solvent uptake.^3−6^ Polarity variation will derive from the chemical structure of monomers and reactions during curing. Free volume relationships are more complex—both monomer structure and the curing process have significant implications.^7−10^

The influence of subtler changes upon network properties is an area of increasing interest.^11−18^ These subtler changes have also been shown to cause variation in properties/chemical performance—from changes to the cure schedule^11,12^ to changes in atmosphere of cure (processing)^19^ and cure method.^20^ Therefore, when attempting to draw comparisons, for changes between monomers, a standardized cure schedule should be used, to avoid variability due to processing.

There are a wide range of possible epoxide and amine combinations which give rise to a range of different properties and performance. A number of studies have shown the impact that subtle changes in monomers can have—for example, Frank and Wiggins present a comparison of 3,3′- and 4,4′-diaminodiphenylsulfone-based networks which shows a 40 °C increase in *T*_g_ for the para-substituted 4,4′-species when cured with DGEBA, accompanied by an increase in water sorption.^12^ In the same study, a shift in *T*_g_ and performance is also observed between the diglycidyl ethers of bisphenol A and F (DGEBA/DGEBF)—with lower *T*_g_’s for the DGEBF species alongside a decrease in water sorption. The same trends in *T*_g_ and water sorption were observed by Alessi and co-workers for DGEBA- and DGEBF-based networks.^21^ Further, epoxy resins cured with the regioisomers of phenylenediamine were shown by Riad et al. to have different *T*_g_’s—the maximum achievable *T*_g_ was found to be highest in the para-isomer, then the meta- and lowest in the ortho-.^22^ Work by the current authors also shows the importance of regioisomerism—networks from the three regioisomers of DGEBF (para–para (pp), para–ortho (po), ortho–ortho (oo)) showed considerable changes in properties and performance, where ortho-substitution was shown to reduce *T*_g_ and to be detrimental to chemical performance.^23^ Recent work by Varley and co-workers also shows changes in properties due to regioisomerism and subtle structural differences.^13−17^ In terms of *T*_g_, they show that para-substitution (relative to meta-) in epoxy resins increases network rigidity and *T*_g_, even where the changes are subtle.

This current work seeks to contrast both amine and epoxide monomers of comparable structures and observe property and performance variation, with a particular focus upon chemical performance. Three key areas will be explored:

A. Geometry of molecules - edited by the position of substituents on six-membered rings (MXDA vs PXDA; 1,3-BAC vs 1,4-BAC; and varied ratios of ortho-/para-substitution in DGEBF-based resins)
B. Nature of chemistry - by changing the nature of six-membered rings from aromatic to aliphatic (MXDA vs 1,3-BAC; PXDA vs 1,4-BAC) and changing the type of bisphenol (DGEBA-based resins vs DGEBF-based resins)
C. Mass between cross-links - the number of atoms between reactive groups will be varied by adjusting the degree of chain extension in epoxy resins (comparison of DGEBA-based resins, E828 and DER 332)

In order to characterize the chemical performance, solvent sorption measurements will be used. Methanol and ethanol were selected as two model cargoes, which are chemically similar but are different in size, in order to study shorter and longer term sorption, respectively.

## Experimental Section

### Materials

The following materials were used as supplied: Dow Epoxy Resin 354 (DER 354) (Olin Corporation); Epikote 828 (E828) (Delta Resins Ltd.); Araldite PY306 (PY306) (Huntsman International LLC); Dow Epoxy Resin 332 (DER 332), crystal violet solution (0.5% solution in glacial acetic acid), meta-xylylenediamine (MXDA), and 1,3-bis(aminomethyl)cyclohexane (1,3-BAC) (Sigma-Aldrich Co. Ltd.); tetraethylammonium bromide, acetic acid, and potassium hydroxide (Fisher Scientific); para-xylylenediamine (PXDA) and 1,4-bis(aminomethyl) cyclohexane (1,4-BAC) (Tokyo Chemical Industry UK Ltd.); and perchloric acid (0.1 mol L^–1^ solution in acetic acid) (VWR International).

### Titration for Epoxide Equivalent Weight (EEW) of Epoxy Resins

Tetraethylammonium bromide (40 g, 0.19 mol) was dried for approximately 1 h at 100 °C. It was then dissolved with warming in acetic acid (450 cm^3^) and allowed to cool. Eight drops of 0.5% crystal violet solution in acetic acid were added and the mixture neutralized to an emerald green color using 0.1 mol dm^–3^ perchloric acid in acetic acid (∼5 cm^3^). Samples of epoxy resin (∼0.1 g) were dissolved in this mixture and then titrated with 0.1 mol dm^–3^ perchloric acid in acetic acid to the emerald green end-point. The epoxide equivalent weight (EEW) was then calculated from eq 1, with three repeats. Full data associated with the titrations can be found in the Supporting Information.
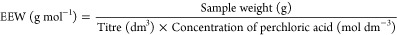
1

### High Performance Liquid Chromatography (HPLC)

HPLC was primarily performed using a Varian Pro Star HPLC system, with a 230 pump and a 310 UV detector. The autosampler used was a 410 model, with a 100 μL injection loop. The column (4.6 × 250 mm^2^) was a Waters XBridge C18 5 μm. The detector was operated at 280 nm, and the system was run at a flow rate of 1.0 mL min^–1^. Samples of ∼1 mg were dissolved in ∼1 cm^3^ of acetonitrile, and injected into the column. A 50:50 acetonitrile:water mixture was used for 19 min, whereupon the system was moved to 100% acetonitrile for a further 10 min.

Due to maintenance, the HPLC analysis for PY306 was performed on a Waters 2695 separation module, with a 2487 dual lambda absorbance detector. The column and method were the same as those used with the Varian system.

### Network Preparation

#### Sample Mixing

Epoxy resin was added to amine in a stoichiometric ratio and then mixed using a stirring rod, covered using parafilm (see Table 1). Each sample was left at room temperature, with occasional mixing (and recovering with parafilm)—we call this time period the pot time. After the pot time, the mixture was sealed and stored overnight at −20 °C in a freezer. This pot time protocol was devised to prevent loss of volatile amine on heating and enable study of the early uptake of solvent (i.e., less than 12 h). The epoxy–amine mixture was then divided for drawdown onto glass slides for uptake measurements, pouring into molds for dynamic mechanical analysis (DMA) and density measurements, and pouring into a glass cell for near-infrared (NIR) cure kinetics measurements.

**Table 1:** Quantities Used in Cures

amine	mass (amine) (g)	moles (amine) (mol)	epoxy	mass (epoxy) (g)	moles (epoxy) (mol)	stoich	pot time (h)
MXDA	2.2584	6.63 × 10^–2^	DER354	11.2228	6.63 × 10^–2^	100.0%	3
1,3-BAC	2.8368	7.98 × 10^–2^	DER354	13.4864	7.97 × 10^–2^	100.1%	2
PXDA^a^	2.9875	8.77 × 10^–2^	DER354	14.8443	8.77 × 10^–2^	100.0%	1.25
1,4-BAC	2.1566	6.06 × 10^–2^	DER354	10.2604	6.06 × 10^–2^	100.0%	2
MXDA	2.7971	8.22 × 10^–2^	E828	15.4733	8.21 × 10^–2^	100.0%	2.5
MXDA	3.0718	9.02 × 10^–2^	PY306	15.0585	9.02 × 10^–2^	100.0%	3
MXDA	2.8835	8.47 × 10^–2^	DER332	14.7063	8.46 × 10^–2^	100.0%	2.5

a PXDA is a solid hardener, and therefore, the pot was held at an elevated temperature (30 °C) in order to reduce the likelihood of crystallization.

### Drawdown onto Glass Slides

The following morning, while sealed (to avoid moisture condensation contaminating the mixture), the vessel was brought to room temperature and then drawn down onto prepared glass slides using a drawdown cube (400 μm slot, Sheen Instruments). The slides were then placed in an oven and cured under the desired atmosphere. For a nitrogen (N_2_) atmosphere, oxygen free nitrogen (BOC) was purged through the oven at ∼5 cm^3^ min^–1^. The cure schedule was an initial temperature of 60 °C, with a ramp of 1 °C min^–1^ to 160 °C, where the oven was held for 3 h. Upon completion of the 3 h, the samples were removed from the oven and allowed to cool. Any samples not immediately analyzed were placed in a desiccator over phosphorus pentoxide to prevent moisture uptake.

### Samples for DMA and Helium Pycnometry

After the pot time, these mixtures were then poured into PTFE molds (for DMA bars), spotted onto release film (for helium pycnometry), and cured. Cure was completed either under a nitrogen or air atmosphere (as for the glass slides). The cure schedule selected ensures a highly controlled cure environment, ensuring the compositions are all treated equally. The nitrogen atmosphere limits side-reactions with air, and the slower ramp rate prevents an unnecessary risk of exotherm.

### Near-Infrared (NIR) Spectroscopy

NIR spectroscopy was performed on an Ocean Optics NIRQuest 2500. Spectra were sampled using an integration time of 10 ms and taking 100 scans to average. Sample cells were prepared by the use of a PTFE spacer between glass slides, which were fastened together using a small amount of epoxy resin.

### Dynamic Mechanical Analysis (DMA)

Dynamic mechanical analysis was performed on a PerkinElmer DMA8000, using single cantilever mode, heated at a rate of 3 °C min^–1^. Three beams were prepared for each sample formulation, approximately 10 mm wide and 1.6 mm thick, and the dynamic response to a sinusoidal force applied at a frequency of 1 s^–1^ recorded. The *T*_g_ was taken as the temperature at the peak in the tan δ trace, from a Lorentzian fit of the peak using OriginLab 2017. A common use of the plateau modulus is to calculate the cross-link density from eq 2.^24^
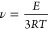
2where *E* is the storage modulus at *T*_g_ + 40 K, *R* is 8.314 J mol^–1^ K^–1^, and *T* is the temperature in K.

This value is provided so as to allow a common metric to be applied allowing comparison between different chemistries in the literature. There is some debate regarding the use of this, as it assumes rubber elasticity theory applies, and these networks are theoretically too highly cross-linked for rubber elasticity to play a significant role. However, a large number of studies dating from the 1960s^25−28^ show that, even for highly cross-linked networks, the calculated value of cross-link density from the plateau modulus is remarkably close to that expected for an ideal network.

### Helium Pycnometry

Helium pycnometry was performed on a Micrometrics AccuPyc 1330, using approximately 0.4 g of sample in a sample cell of 1 cm^3^ and a standard of known mass and volume for calibration.

### Solvent Sorption/Desorption Measurements

Upon completion of cure upon glass slides, the networks were immersed in solvent (methanol/ethanol) and weighed periodically, after removing excess solvent with a paper towel. In methanol, immersion was continued until a plateau in mass was obtained. At this point, the glass slides were placed in an oven at 40 °C and again weighed periodically (Table S2). No plateau was obtained for ethanol sorption, and therefore, desorption measurements were not performed.

## Results and Discussion

### Epoxy Monomer Variation

**Figure 1:**
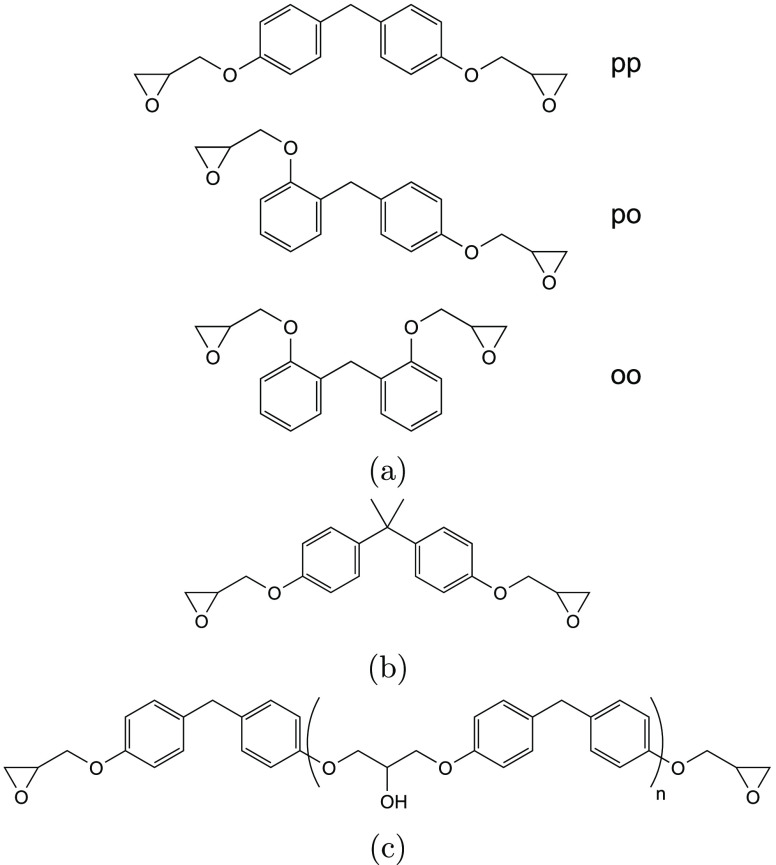
Base structures of epoxy resin monomers: (a) DGEBF (para–para, para–ortho, and ortho–ortho isomers shown); (b) DGEBA and (c) the structure of “chain extended” isomers when only considering para-substitution in DGEBF. DGEBF - diglycidyl ether of bisphenol F; DGEBA - diglycidyl ether of bisphenol A.

The epoxy monomers used in this study vary in three key areas:

(i) The type of bisphenol (see Figure 1a,b)
(ii) The degree of chain extension (quantified by *n*, see Figure 1c)
(iii) The distribution of regioisomers (only for DGEBF-based resins, see Figure 1a)

Characterization of the resin mixtures used is necessary to effectively draw comparisons between the subsequently prepared cross-linked networks.

**Figure 2:**
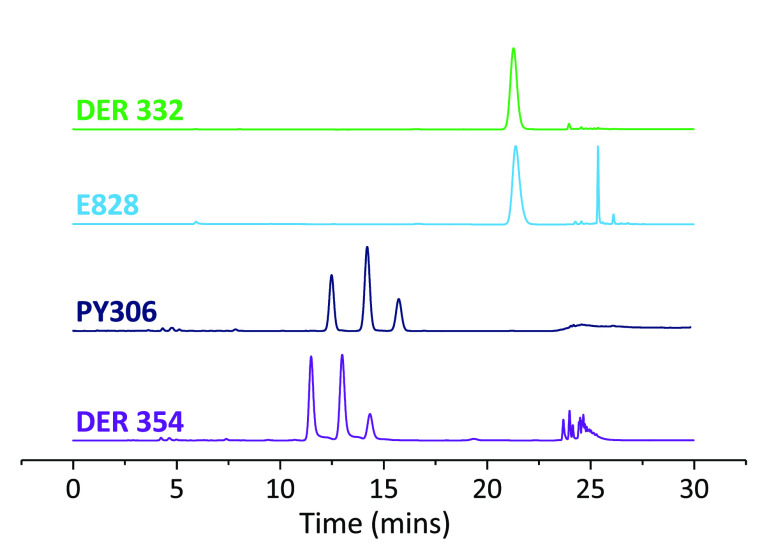
HPLC chromatograms for DER 332, E828, PY306, and DER 354 using a 50:50 acetonitrile:water isocratic solvent system. Relative quantities for the DGEBF resins were calculated by integration of the peaks. The three peaks at 10–16 min are pp, po, and oo in time order, as found by Pontén et al.^29^ and confirmed in our recent work.^23^ Key: DER 332 - Dow Epoxy Resin 332; E828 - Epikote 828; PY306 - Araldite PY306; DER 354 - Dow Epoxy Resin 354. nb. PY306 was run on a different machine due to maintenance issues (using the same column).

HPLC traces for those monomers demonstrate the diversity of chemical species in each resin (Figure 2). DER 332 (a DGEBA-based resin) shows very little diversity, with a single species (Figure 1b) making up the vast majority of the mixture, whereas DER 354 (DGEBF-based) shows a large variety of species (Figure 1a and c, with *n* = 0, 1, 2, ...). As discussed earlier (at the end of the Introduction), in DGEBF-based resins, there are three regioisomers (pp/po/oo) which are represented by the three peaks between 10 and 17 min for the DGEBF-based resins (DER 354/PY306). The single peak at ∼20 min in the (DGEBA-based) E828 and DER 332 traces represents the one regiosomer present for DGEBA. Any peaks after 23 min are related to chain extended structures. It is apparent that the diversity of chain extended isomers is much greater again for DGEBF-based resins when observing the DER 354 trace. This is because reaction between bisphenol units can be between any two regioisomers, and for the asymmetric po isomer, addition can be to either end. This leads to nine different possible *n* = 1 isomers. Since there is only one regioisomeric motif present in DGEBA, there is only one *n* = 1 isomer that is observed in the E828 trace.

^1^H NMR spectroscopy (for spectra, see the Supporting Information) also shows evidence of regioisomerism for the DGEBF-based resins, according to the method described by Domke.^30^ Both ^1^H NMR and HPLC provide a route to obtaining the isomeric ratio (by integration of the relative peaks). *[nb. PY306 was run on a different machine due to maintenance issues (using the same column); therefore, comparison of absolute values is not possible—the analysis here only used peak order]*. It is worth noting that, while HPLC provides a simple ratio of the three *n* = 0 isomers, NMR provides a ratio of the regioisomerism in the material as a whole (including chain extended structures). (HPLC is not used for the chain extended isomers, since while it is reasonable to assume absorption of UV light is very similar for the *n* = 0 regioisomers, higher MW species may absorb differently, due to their more significantly altered chemical structure, and so out of caution comparison is avoided here.) In any case, it is clear from both techniques that DER 354 contains a higher proportion of the pp isomer relative to PY306. ^1^H NMR spectroscopy also provides a route to a measurement of the epoxide equivalent weight (EEW), which in turn can be used to predict the degree of chain extension—quantified by an average value for *n* for the chemical structure shown in Figure 1c.^31^ This relies on the assumption that there are two epoxide groups per molecule. Titration has also been used to find EEW. The combined results from the analytical methods described are shown in Table 2. It is worth noting that, while there is only a minimal signal for high MW species in PY306, the measured epoxide equivalent weight corresponds to a degree of chain extension for which a much greater signal would be expected. This suggests that the assumption of two reactive groups per molecule may not be applicable; i.e., the resin has a slightly reduced functionality to that expected.

**Table 2:** Degree of Chain Extension (See Figure 1c) with the Corresponding Epoxide Equivalent Weight in Parentheses and Regioisomeric Distributions (pp:po:oo; See Figure 1a for Structures) for the Epoxy Resins Studied Obtained by Titration and ^1^H NMR and HPLC and ^1^H NMR, Respectively^a^

		*n* (EEW/g mol ^–1^)		isomeric ratio	
resin	basis	titration	NMR	HPLC	NMR
DER 354	DGEBF	0.10 (169)	0.10 (169)	40:45:15	47:40:13
PY306	DGEBF	0.08 (167)	0.07 (165)	30:50:20	36:49:14
E828	DGEBA	0.13 (188)	0.13 (188)	n/a	
DER 332	DGEBA	0.02 (174)	0.01 (172)	n/a	

a For more details on how values were obtained, see the Supporting Information.

**Figure 3:**
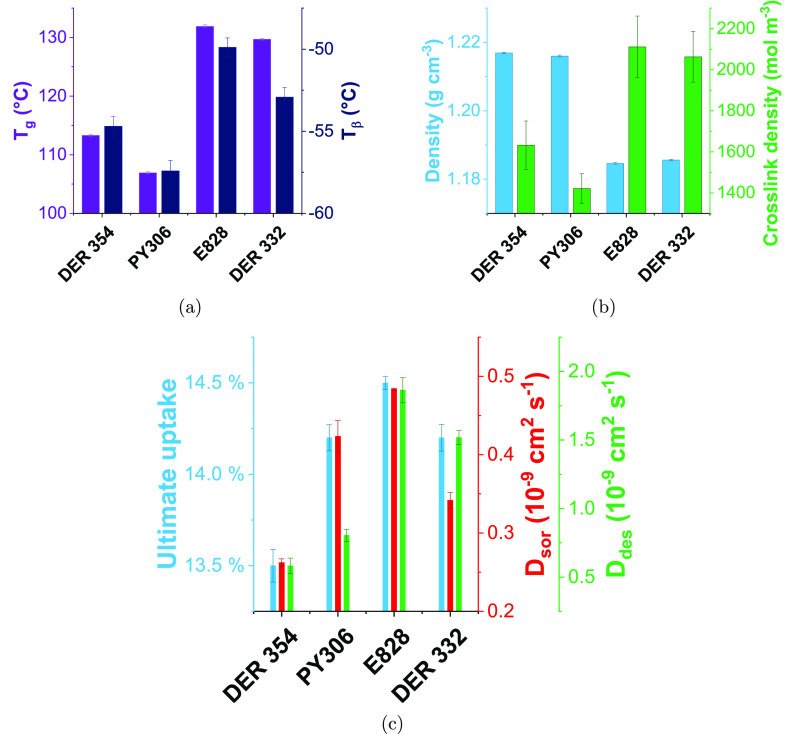
(a) *T*_g_ and *T*_β_ as measured by DMA and (b) density as found by helium pycnometry and cross-link density (also found by DMA from the rubbery plateau modulus) of networks prepared with varied epoxy resin monomers and MXDA. The error bars show the standard error of 3 measurements for the values obtained by DMA and the standard deviation of 10 measurements of a single sample for density. (c) Summary of methanol uptake parameters (the ultimate uptake and diffusion coefficients for sorption (*D*_sor_) and desorption (*D*_des_)) for networks made with a range of epoxy resin monomers and MXDA. The error bars show the standard error of three samples.

Each of the epoxies were cured using meta-xylylenediamine (MXDA)—Figure 3 shows properties obtained from dynamic mechanical analysis, helium pycnometry, and solvent sorption/desorption of the resulting networks.

### DGEBF-Based Resins

**Figure 4:**
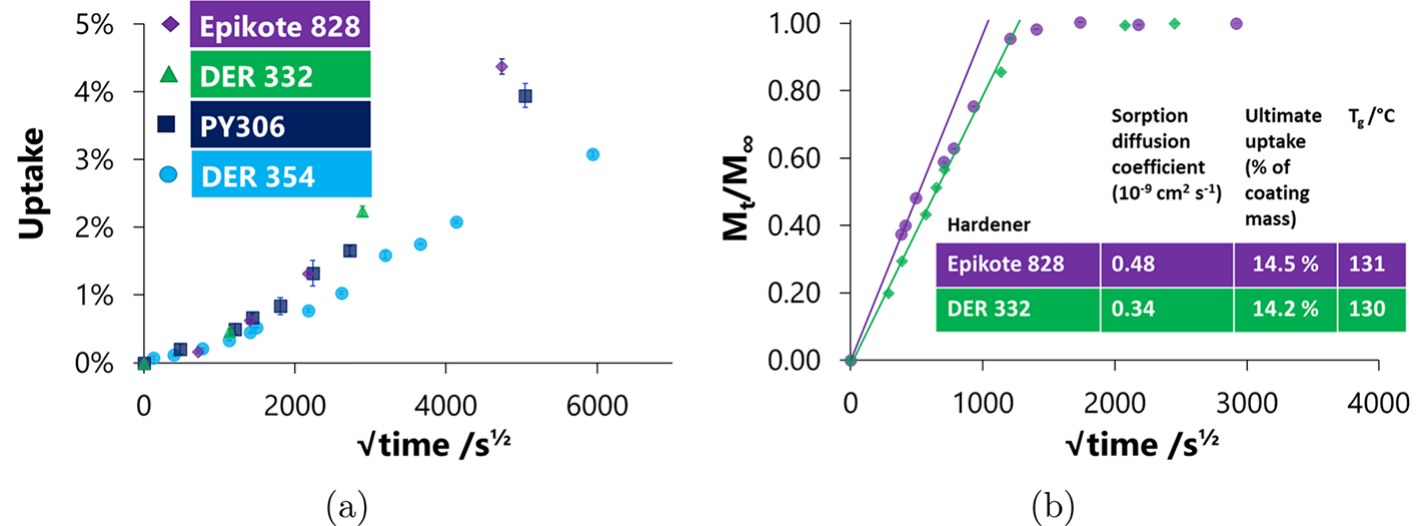
(a) Ethanol sorption of networks on glass slides made with varying epoxy resin monomers and MXDA. (b) Methanol uptake curves for (the DGEBA-based) E828 and DER 332 samples cured with MXDA. Initial slopes are projected to . The trace for E828 is seen to deviate from the initial slope at a much lower  than the curves observed for DER 332.

Comparing the DER 354 and PY306 shows similar mass density, as would be expected with their similar EEW, though there is a reduction in the *T*_g_, *T*_β_, and cross-link density for the PY306. This suggests, but is not definitive proof, that it is the variation in the ortho-content that limits the cross-linking and hence chain mobility (*T*_g_, *T*_β_).^23^ This is accompanied by an increase in methanol uptake both in terms of rate and ultimate uptake. Further, the ethanol uptake profiles (Figure 4a) show reduced uptake for DER 354 relative to PY306. In both cases, a plateau in sorption was not obtained in spite of long uptake time, and the sorption is sigmoidal (i.e., there is an upturn in the gradient of  vs ) indicating swelling is present.^32,33^ The major chemical differences between these resins is the regioisomeric composition and varied degrees of chain extension (see Table 2 for *n* and EEW values). There is also some suggestion of a slightly reduced functionality for PY306, though near-infrared results are suggestive of complete reaction of epoxide groups (see the Supporting Information). The current authors have previously shown that ortho-content is detrimental to chemical performance,^23^ and indeed the PY306 contains a higher proportion of ortho-substitution (45% of aromatic rings vs 37.5% for DER 354). However, the magnitude of the changes in properties observed is suggestive that the qualitatively observed functionality reduction also plays a part. In any case, the variation shows the importance of chemical analysis of DGEBF-based resins in understanding structure–property relationships and selecting an appropriate resin for a particular application.

### DGEBA-Based Resins

Where E828 and DER 332 are compared, networks with similar thermal/physical properties are observed, but a more marked variation in chemical performance is seen (Figure 3c). A slight reduction in both *T*_g_ and *T*_β_ is observed for DER 332 (vs E828), accompanied by a slight density increase. Here there is a complete absence of pp:po:oo isomerism and only the EEW differs. This adds weight to the above argument that EEW has little influence on *T*_g_ and *T*_β_ within this EEW range. Ultimate uptake and *D*_sor_ are both observed to decrease more than might be expected with the similarity in thermal/physical properties. The profile of sorption is also of interest—it is observed in Figure 4b. DER 332 shows classical Fickian uptake, though the uptake for E828 is of the two-step mode, which is suggestive of some degree of heterogeneity in the network.^32,33^ An initial steeper uptake of methanol is followed by a discontinuity and a region of slower uptake. A possible explanation could be the presence of two solvent environments within the E828 network which is absent in the DER 332—created by the presence of chain extended isomers. The presence of the linear chain extension, found in E828, results in topographic (linear versus cross-linked) and chemical (aromatic rings versus polar) differences. If the solvent uptake in these two regions is different (we propose that the first of these environments may be more accessible than the second), this would lead to the steeper gradient initially observed. This is supported by the gradient of the second region of uptake resembling the initial uptake of DER 332. The influence of molecular motion will be discussed in more detail when comparing 1,3- and 1,4-substitution in amines below, but the increased peak area for the β-transition of E828 when compared to DER 332 may provide some justification for faster uptake—since molecular motion may enhance solvent uptake (see Figure SI8 in the Supporting Information for β-transition data). Ethanol uptake showed limited variation between E828 and DER 332.

### Comparison of DGEBA- and DGEBF-Based Networks

**Figure 5:**
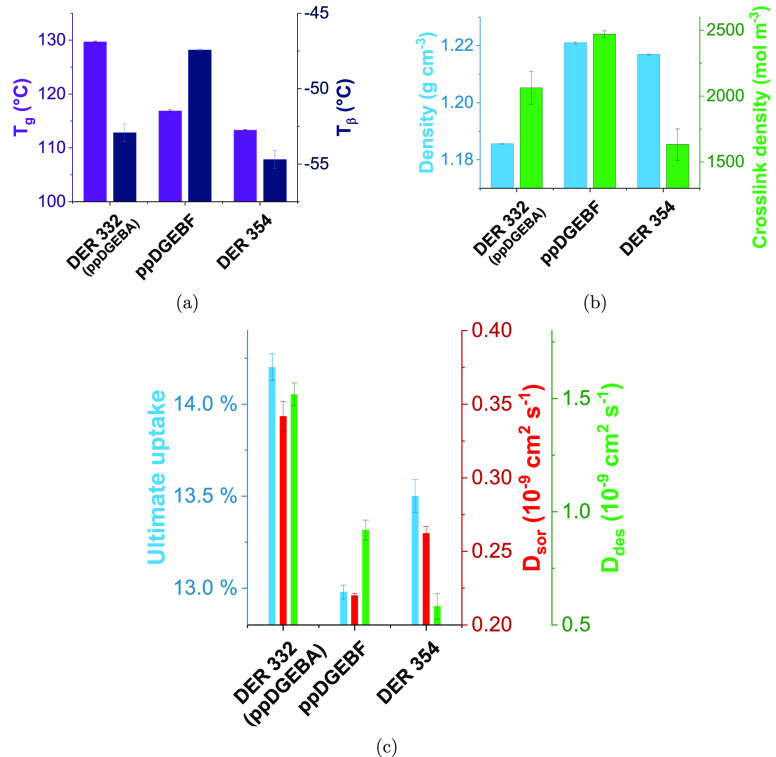
(a) *T*_g_ and *T*_β_ as measured by DMA and (b) density as found by helium pycnometry and cross-link density (also found by DMA from the rubbery plateau modulus) and (c) methanol uptake properties for ppDGEBF, DER 332 (ppDGEBA), and DER 354. Part c shows the ultimate uptake and diffusion coefficients for sorption (*D*_sor_) and desorption (*D*_des_) of methanol.

The DGEBF-based networks were shown to be denser than the DGEBA-based networks, in spite of a reduction in measured cross-link density. The increased density is likely derived from the reduction in packing efficiency caused by the presence of the methyl groups present on the bridging carbon in DGEBA. Regarding cross-link density, it is worth noting that using DMA to determine cross-link density relies upon a contribution to the mechanical strength from each segment between cross-links.^24^ The molecular diversity of DGEBF-based resins means the likelihood of such a theory holding true is not high, and may provide a justification for the difference in the values obtained. Mixed trends in chemical performance were observed, though the best-performing DGEBF-based system had lower and slower methanol sorption than its DGEBA-based counterpart. Further, ethanol uptake was reduced for the DGEBF-based networks. In order to directly measure the influence of the methyl groups present in DGEBA that are not present in DGEBF (as shown in the structures in Figure 1), purer networks are necessary. Previous work by these authors showed isolation of ppDGEBF,^23^ and a comparison of the properties and methanol performance of this and the DER 332 (which is effectively ppDGEBA) are shown in Figure 5. The comparison again shows the DGEBF to be denser in spite of a lower *T*_g_, though the cross-link density is much increased relative to the DER 354, and is higher than DER 332—supporting the earlier suggestion that DER 354 contains a range of mechanically inactive segments which are still space-filling and therefore improve chemical performance. There is a clear reduction in the rate and amount of methanol sorption (Figure 5c). This shows the presence of the bridging methyl groups is detrimental to the packing and subsequent performance of epoxy resins—indeed, this is supported by the improved performance of DER 354 (relative to DER 332) in spite of the molecular diversity and increased chain extension (which was shown by E828 vs DER 332 to be detrimental).

### Amine Monomer Variation

**Figure 6:**
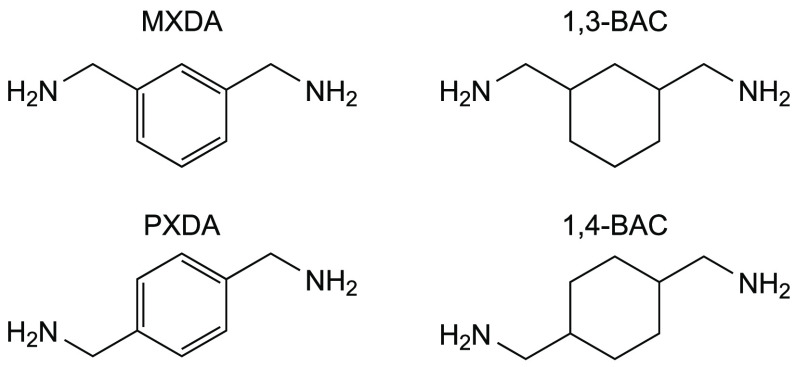
Amines used in this study. Key: MXDA - meta-xylylenediamine; 1,3-BAC - 1,3-bis(aminomethyl)cyclohexane; PXDA - para-xylylenediamine; 1,4-BAC - 1,4-bis(aminomethyl)cyclohexane; PACM - bis(para-aminocyclohexyl) methane.

The amines studied are shown in Figure 6 and allow us to explore the property/performance changes imparted by aromatic/aliphatic bonding changes and 1,3-/1,4-substitution changes. It is important to use structures with the methylene spacer groups to solely study the impact of changing aromaticity upon packing, since aromatic amines (those directly bound to the ring) show different kinetic properties to aliphatic analogues and therefore give rise to different network growth kinetics.^34^ We do not discount the possibility of smaller variations in kinetics, but the approach taken will mitigate the effects for the most part. Each network was cured with DER 354 in a stoichiometric ratio and showed >99% conversion by NIR spectroscopy (see Table SI4 in the Supporting Information).

### Aromatic/Aliphatic Comparison

**Figure 7:**
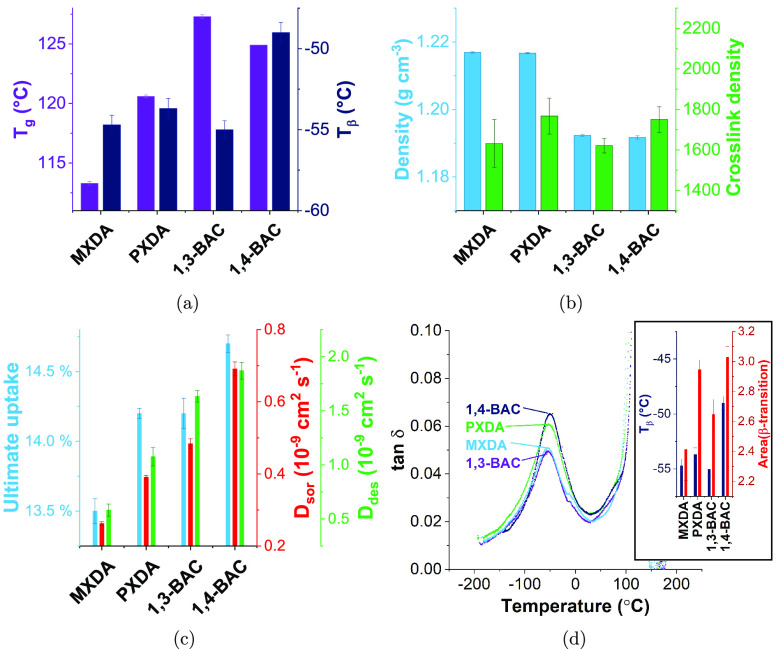
(a) *T*_g_ and *T*_β_ as measured by DMA and (b) density as found by helium pycnometry and cross-link density (also found by DMA from the rubbery plateau modulus) of the four different networks prepared with DER 354 and varied amine hardeners. The error bars show the standard error of 3 measurements for the values obtained by DMA and the standard deviation of 10 measurements of a single sample for density. (c) Summary of methanol uptake parameters for networks made with a range of amines and DER 354. The error bars show the standard error of three samples. (d) DMA traces of tan δ against temperature for networks prepared with varied amine hardener, showing the response for the β-transition. Inset: The *T*_β_ and area of the β-transition for those networks. The error bars show the standard error of two samples.

**Figure 8:**
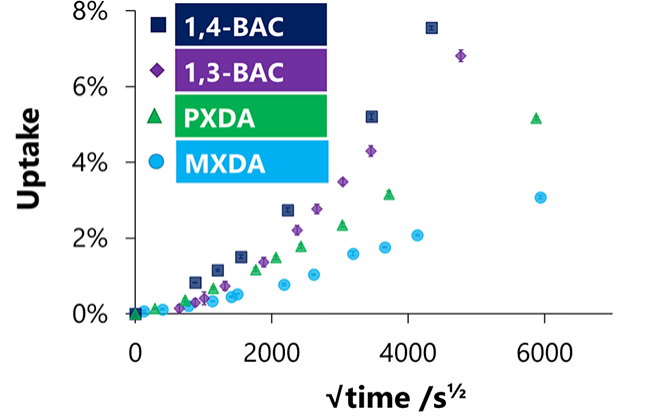
Ethanol uptake for networks on glass slides made with varying amines and DER 354.

Figure 7 shows some clear differences between aromatic and aliphatic networks. While the aromatic networks are shown to be more dense, they are also, perhaps unexpectedly, shown to have lower *T*_g_’s than their aliphatic analogues. Generally, aromaticity is associated with a rigid polymer backbone and hence a higher *T*_g_.^35^ This unusual relationship supports work by Ochi et al. which shows the same trend with MXDA and 1,3-BAC cured with E828.^36^ Where direct comparisons between the aromatic networks and their aliphatic analogues are drawn (i.e., MXDA with 1,3-BAC and PXDA with 1,4-BAC), there is a marked increase in both the rate and amount of methanol and ethanol sorption (Figures 7c and 8) for the aliphatic samples. Ethanol uptake was again sigmoidal (indicating swelling processes) for all networks, but the increase in gradient in  vs  was far greater for the aliphatic systems. These data show the more effective packing of the aromatic networks when compared to aliphatic analogues. While stiffer polymers do generally pack less efficiently, and indeed high intrinsic porosity is seen in the very stiff ladder polymers, here we have molecular liquids that react together to create a network.^37^ The network structure is effectively determined at the gel point of the network, where there is still considerable molecular mobility. The bent cyclohexyl aliphatic structure fills space significantly less efficiently than the flat aromatic rings, leading to a greater degree of free volume existing in the material permanently (as is supported by the density measurements). Stacking of either ring structure (aliphatic or aromatic) is highly unlikely in the amorphous material.

### 1,3-/1,4-Substitution

Similar trends in chemical performance were observed with the move from 1,3- to 1,4-substitution for both aromatic and aliphatic systems; that is, there is an increase in the rate and amount of methanol and ethanol sorption. However, the changes in *T*_g_, density, or cross-link density do not provide a justification for this and indeed the changes are not necessarily similar. While the *T*_β_ shows some variation, Figure 7d shows an increased peak area for the β-transition for the 1,4-substituted structures, indicating the presence of a molecular motion absent from the 1,3-substituted structures. The inclusion of meta-substituted structures has been shown to improve chemical performance (i.e., reduced solvent sorption) and reduce gas permeability.^12,38^ Furthermore, an increase in density and the aforementioned reduction in molecular motion was observed by Ramsdale-Capper and Foreman (for meta-containing networks), in conjunction with a range of improvements in desirable mechanical properties such as strain to failure and fracture toughness.^39^ Since diffusion in polymers is dependent on kinetic coefficients for both solvent and polymer, it follows that this reduced motion could lead to improved chemical performance.^32^

## Conclusions

With regard to the previously identified three key areas of investigation, significant variation in chemical performance can be obtained for small changes in (A) molecular geometry, (B) the nature of chemistry, and (C) the distance between cross-links. In particular for molecular geometry, 1,3-substitution in amine monomers is shown to be beneficial for performance (relative to 1,4-) in spite of unexpected corresponding trends in thermal/physical properties. Reduced small-scale molecular motion observed in the β-transition for 1,3-substituted structures may help explain this reduction in ultimate uptake and diffusion rates.

From the two notable adjustments in chemistry (without structural rearrangement), aromatic monomers produced denser, better performing networks than their aliphatic analogues, with lower and slower uptake of methanol and ethanol. DGEBF-based networks generally also show improved chemical performance relative to DGEBA-based networks (again accompanied by an inverse trend in *T*_g_), with a greater density. However, the worse performing industrial mixture of DGEBF, PY306, showed a significant reduction in performance—which can be explained by (a) an increased ortho-content and (b) a reduced functionality. Cross-link density measurement for industrial DGEBF-based mixtures showed a significant reduction relative to the isomerically pure ppDGEBF, indicating that a great deal of the linkages between cross-links are not mechanically active, though the similar chemical performance of DER 354 and ppDGEBF shows these parts of the network still contribute to resisting solvent uptake. When comparing like-for-like (i.e., isomerically pure ppDGEBA and ppDGEBF, with very limited chain extension), the difference in properties and performance is most pronounced. Finally, an increase in the average distance between cross-links was shown to be detrimental to chemical performance, as shown by the two DGEBA-based resins, where DER 332 showed better performance than E828.

The amalgam of factors which influence chemical performance in epoxy networks mean that a single individual property cannot be used as a universal qualifier of chemical performance, though generally increased density and reduced mobility (as indicated by the area of the β-transition) were indicative of better performance. Future work to deepen the understanding of why interactions between chemicals and the network differ could include further broadening of the search area—whether by diversifying the basis of the network or the solvents studied.

## Supporting Information Available

Characterization data, including the data processing methodology and a summary table for all properties for the networks prepared in this work (PDF)
