# Supplementary material for: Structural Variation and Chemical Performance—A Study of the Effects of Chemical Structure upon Epoxy Network Chemical Performance
Source: ACS Appl Polym Mater. 2021 Jun 15;3(7):3438–45. doi: 10.1021/acsapm.1c00378 (PMC8291510; doi:10.1021/acsapm.1c00378)
Supplement: Supplementary file 1 — ap1c00378_si_001.pdf [file ap1c00378_si_001.pdf]

Supporting information (SI) for:

Structural variation and chemical performance – a study of the effects of chemical structure upon epoxy network chemical performance.

Stephen T. Knox[*,†,¶], *Anthony Wright*[‡], *Colin Cameron*[‡], *John Patrick Anthony Fairclough*[*,†]

†Department of Mechanical Engineering, University of Sheffield, Sheffield S1 4BJ, UK.

‡AkzoNobel, International Paint Ltd, Stoneygate Lane, Gateshead, NE10 0JY, UK

¶Current address: School of Chemical and Process Engineering, University of Leeds, LS29JT, UK

E-mail: s.knox@leeds.ac.uk; p.fairclough@sheffield.ac.uk

Contents

NMR Spectroscopy 3

Titration for epoxide equivalent weight (EEW) of epoxy resins 7

NIR spectroscopy 8

Dynamic mechanical analysis 11

Solvent sorption/desorption 14

Summary table 16

References 17

NMR Spectroscopy

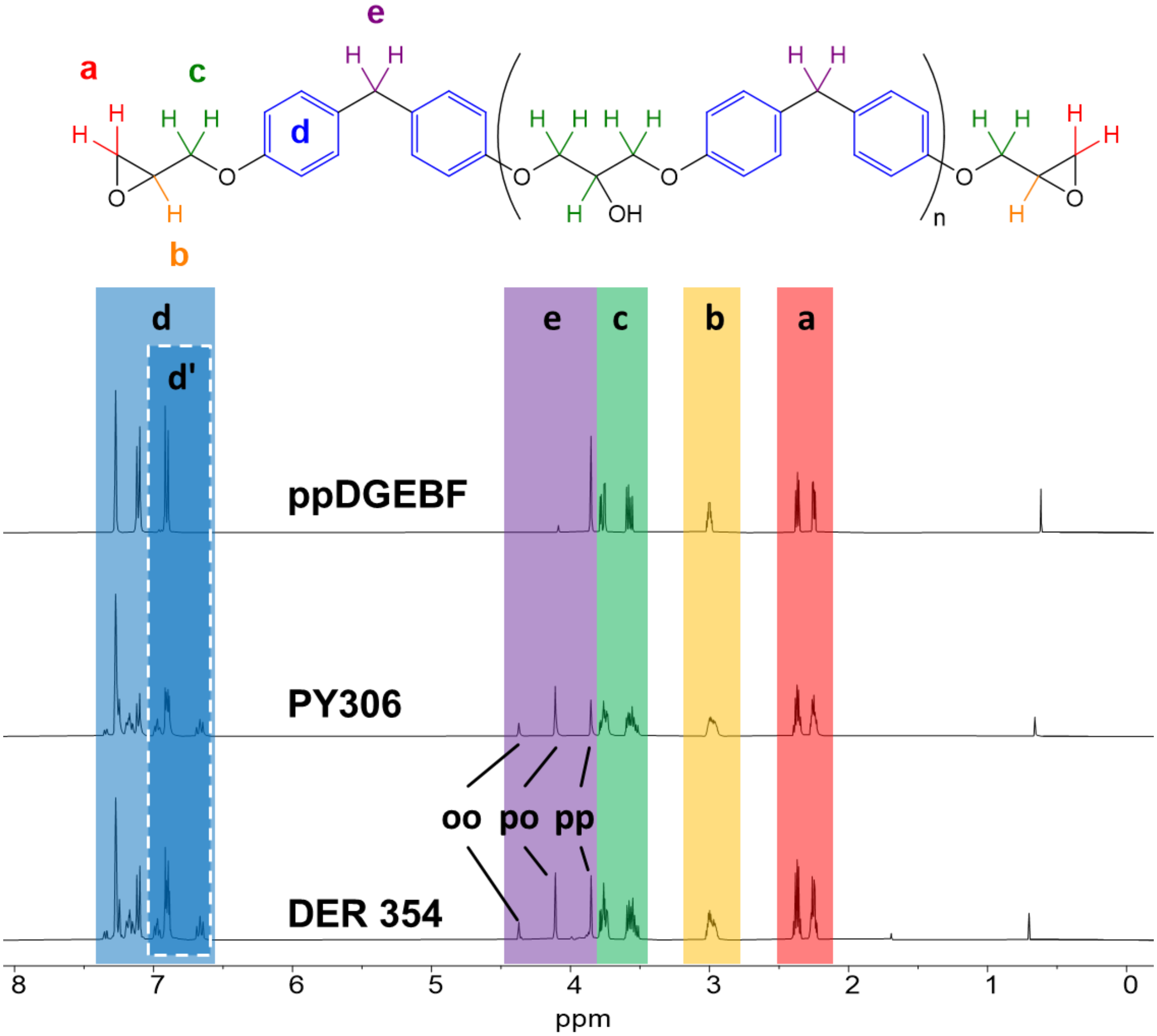

Figure SI1. ^{1}H NMR spectra for DGEBF based resins used in this work. Generalised assignments are also shown.

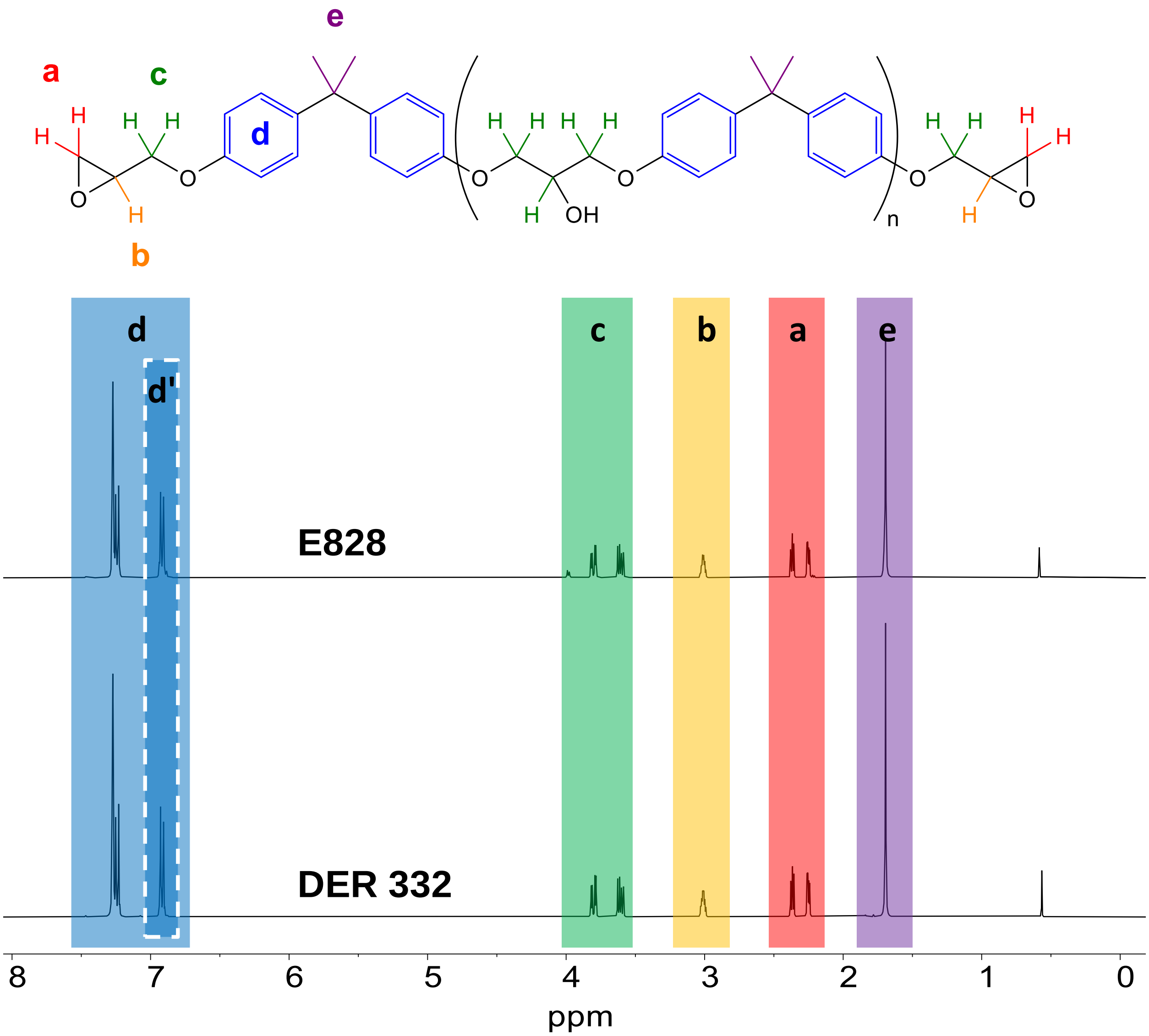

Figure SI2. ^{1}H NMR spectra for DGEBA based resins used in this work. Generalised assignments are also shown.

Figures SI1 and SI2 show the ^{1}H NMR spectra obtained for the epoxy resin monomers used in this work. Note for DGEBF based resins the complexity of the spectra for the industrial mixtures (PY306/DER 354) vs. the isomerically pure ppDGEBF. Degree of chain extension was determined by comparing the integrals for the phenyl protons not obscured by solvent (2H, d′) and the terminal

epoxide protons (3H, a/b) – more details are given in Table SI2. The epoxide equivalent weight can be determined using Equation SI1.

$$EEW = \frac{(n \times MW_{n\,segment}) + MW_{n=0}}{f}$$

Equation SI1. The epoxide equivalent weight (EEW) from the average degree of extension (n) and functionality (f) of a given resin, and the molecular weights of the n = 0 molecule ($MW_{n=0}$) and the n segment of that resin ($MW_{n\,segment}$) (see Table SI1)

Table SI1. The $MW_{n=0}$ and $MW_{n\,segment}$ for DGEBA and DGEBF based resins.

Resin basis	$MW_{n=0}$ /g mol^{-1}	$MW_{n\,segment}$ /g mol^{-1}
DGEBF	312.36	256.31
DGEBA	340.42	284.36

Table SI2. Integrals from selected regions of the ^{1}H NMR spectra for the epoxy resins used in this work and derived values for the isomeric ratios of isomers in DGEBF and n, the average degree of chain extension.

	**Integrals**									**Calculated values**	
	**d'**<br>6.89-6.42 (for DGEBF)<br>6.88-6.70 (for DGEBA)	**e_{oo}**<br>4.41-4.35	**e_{po}**<br>4.17-4.08	**e_{pp}**<br>3.91-3.82	**c_i**<br>3.76-3.60	**c_{ii}**<br>3.56-3.41	**b**<br>2.97-2.82	**a_i**<br>2.31-2.20	**a_{ii}**<br>2.17-2.11	**pp : po : oo ratio**<br>$\frac{e_x}{e_{oo}+e_{po}+e_{pp}}$<br>for each isomer	**n**<br>$\frac{d'}{2} - mean(a_i, a_{ii}, b)$
DER 354	2.25	0.15	0.48	0.52	1.09	1.09	1.00	1.03	1.03	0.45 : 0.41 : 0.13	0.10
PY306	2.16	0.16	0.53	0.39	1.11	1.10	1.00	1.02	1.02	0.36 : 0.49 : 0.14	0.07
ppDGEBF	2.11	0.00	0.00	1.08	1.05	1.05	1.00	1.03	1.03	1 : 0 : 0	0.03
E828	2.30		n/a		1.08	1.08	1.00	1.03	1.02	n/a	0.13
DER 332	2.08				1.06	1.07	1.00	1.03	1.05		0.01

Titration for epoxide equivalent weight (EEW) of epoxy resins

Full details of the experimental procedure can be found in the manuscript itself. Table SI3 shows the results for each resin in this work. EEW is calculated from Equation SI2. n is calculated by rearranging Equation SI1.

Table SI3. Data for titrations performed to determine EEW for the resins in this work.

Sample name	Run	Mass (epoxy) used / g	Start volume /cm^3	End volume /cm^3	Titre /cm^3	EEW /g mol^{-1}	Average EEW /g mol^{-1}	n
DER 354	1	0.1143	0.02	6.77	6.75	169.3	169.2	0.10
	2	0.1035	0.2	6.33	6.13	168.8		
	3	0.1296	0.36	8.01	7.65	169.4		
PY306	1	0.1046	0.02	6.27	6.25	167.4	166.9	0.08
	2	0.1103	0.04	6.64	6.6	167.1		
	3	0.1186	0.02	7.16	7.14	166.1		
ppDGEBF	1	0.1008	0.74	6.92	6.18	163.1	162.3	0.05
	2	0.1019	0	6.28	6.28	162.3		
	3	0.1024	0.02	6.36	6.34	161.5		
E828	1	0.1017	0	5.4	5.4	188.3	188.4	0.13
	2	0.1001	0	5.32	5.32	188.2		
	3	0.1141	0	6.05	6.05	188.6		
DER 332	1	0.1114	0	6.42	6.42	173.5	173.8	0.02
	2	0.1076	0.04	6.22	6.18	174.1		
	3	0.1087	0.04	6.3	6.26	173.6		

$$\mathrm{EEW}(\mathrm{g\ mol^{-1}}) = \frac{\mathit{Sample\ weight}\ (g)}{\mathrm{Titre\ (dm^3)} \times \mathrm{Concentration\ of\ perchloric\ acid\ (mol\ dm^{-3})}}$$

Equation SI2. Calculation of EEW from titration data.

NIR spectroscopy

The following description of NIR spectroscopy can also be found in our previous work's[1] supplementary information, but is included here for ease of access:

The epoxide peak at 4529 cm^{-1} was shown to decrease as epoxy-amine reaction progressed (**Figure SI3**), to complete disappearance at full conversion. However, the absorbance intensity did not go to zero due to the presence of strong neighbouring peaks. Therefore, it was not appropriate to use peak intensity as a measure, and instead a local fit of the six peaks between 4340 and 4660 cm^{-1} was performed using OriginLab 2018b and the integral of the fitted peaks used for absorbance (preferred to direct integration of peaks due to the integral range being a trough in the final product).

The extinction coefficient for the epoxide peak at 4529 cm^{-1} was determined using varied path thicknesses of DER 354, and then performing a linear fit on the plot of absorbance (i.e. fitted peak integral of absorbance) vs. path length. Using this method, the extinction coefficient for epoxide at 4529 cm^{-1} was determined to be 43.27 kg mol^{-1} cm^{-1} or 36.36 dm^3 mol^{-1} cm^{-1}. This is similar to the values found in the literature: Jackson et al. found it to be 36.64 dm^3 mol^{-1} cm^{-1}, Sahagun et al. found it to be 58.8 kg mol^{-1} cm^{-1} and Poisson et al. found it to be 34.9 kg mol^{-1} cm^{-1}.[2–4]

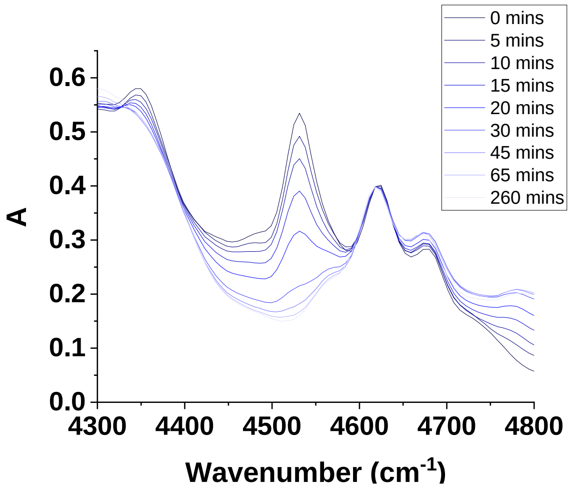

Figure SI3. Evolution of NIR spectra over time of a reacting mixture of ppDGEBF and MXDA at 160 °C. The disappearance of the epoxide peak is observed at 4529 cm^{-1}.

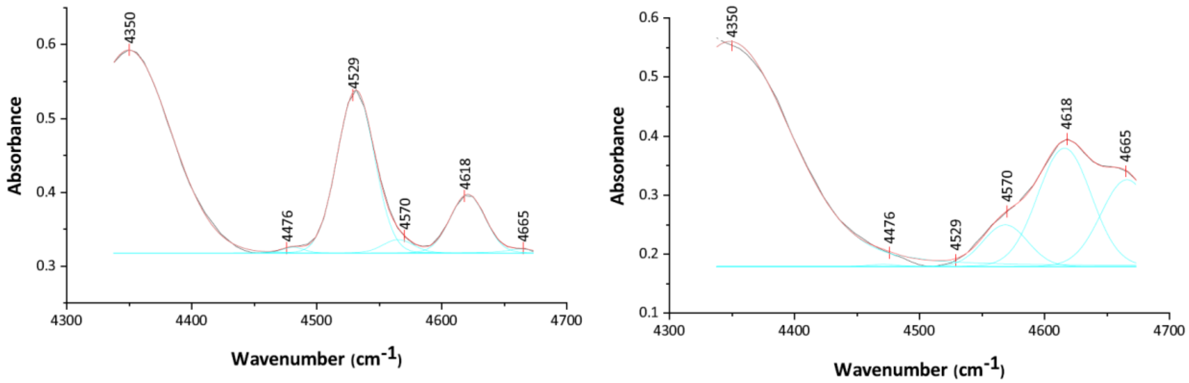

Figure SI4. Fitting of the peaks including and surrounding the epoxide peak (4529 cm^{-1}) for (a) the initial scan (t = 0 mins) and (b) the final scan (t = 260 mins) in Figure SI3.

To enable the calculation of conversion (Equation SI3) at any point during the reaction, we use the mass extinction coefficient, as is common in the literature – since the initial concentration can be calculated from formulation. The concentration at any time is calculated using Equation SI4, using the peak area for A.

$$\alpha = 1 - \frac{C_t}{C_0}$$

Equation SI3. Conversion, α, where C_t is the concentration at time = t and C_0 is the initial concentration

$$C = \frac{A}{\varepsilon l}$$

Equation SI4. Beer-Lambert law where C is concentration, A is the absorbance, ε is the extinction coefficient and l the path length.

Dynamic mechanical analysis

Experimental details for the DMA performed in this work can be found in the Experimental of the main manuscript. Figures SI3-5 show the raw traces for the modulus and tan δ with temperature.

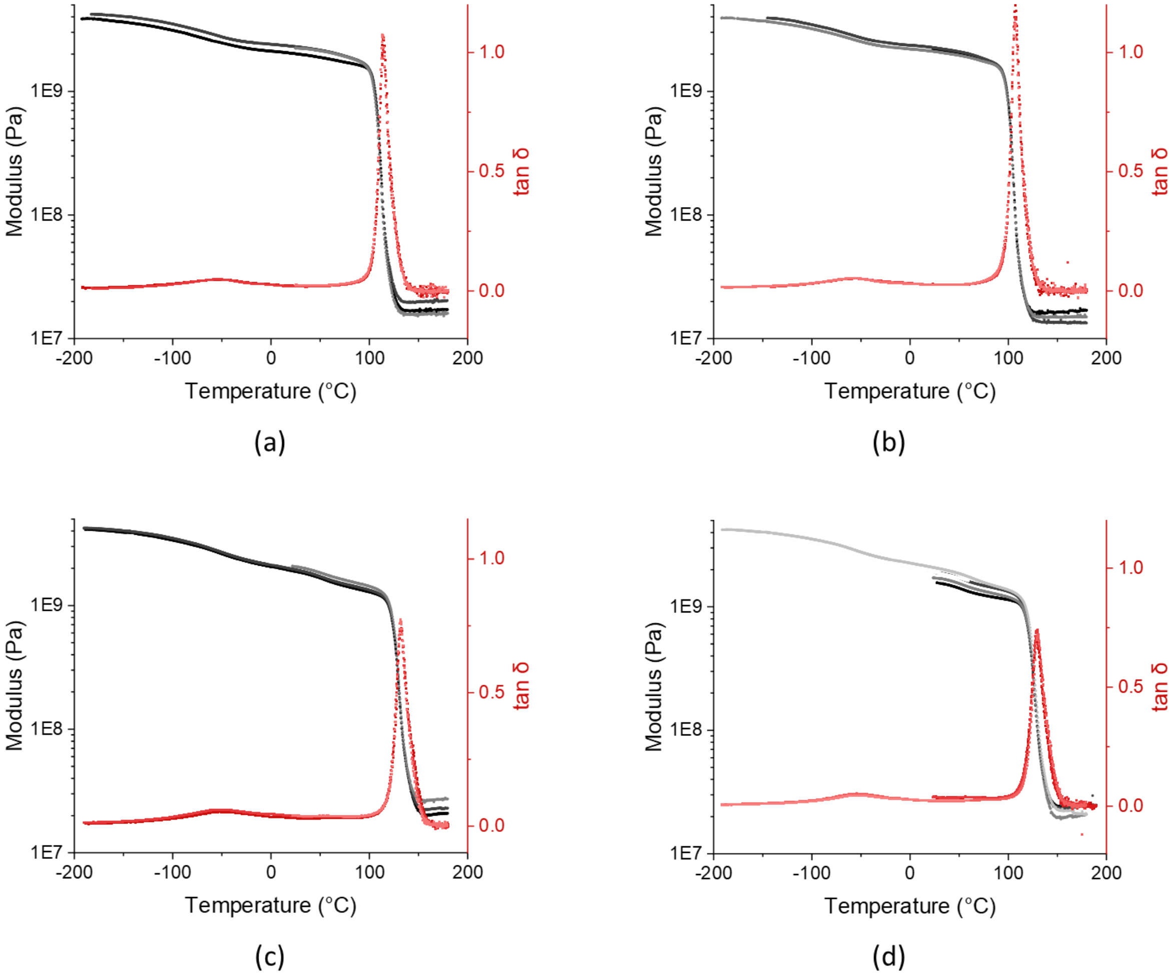

Figure SI5. Dynamic mechanical analysis for the four networks made with MXDA and (a) DER 354, (b) PY306, (c) E828 & (d) DER 332

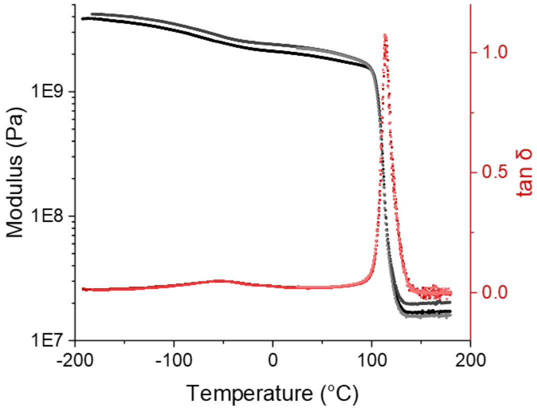

(a)

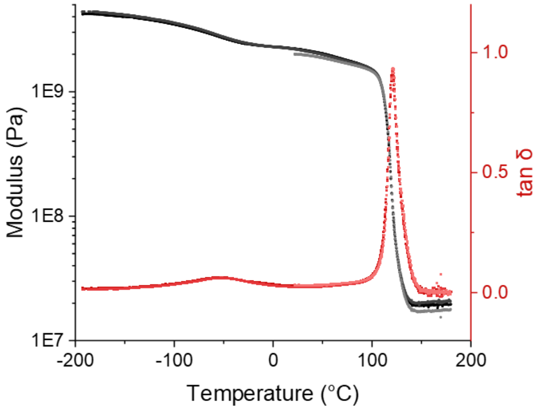

(b)

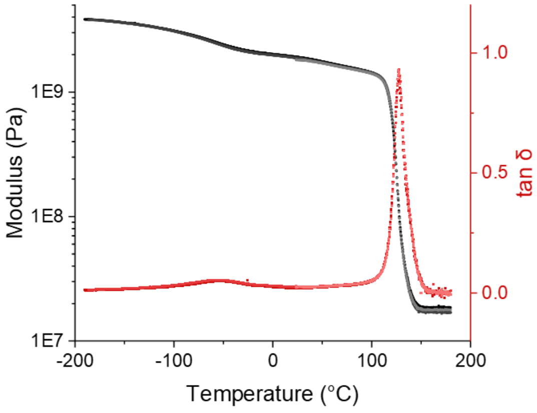

(c)

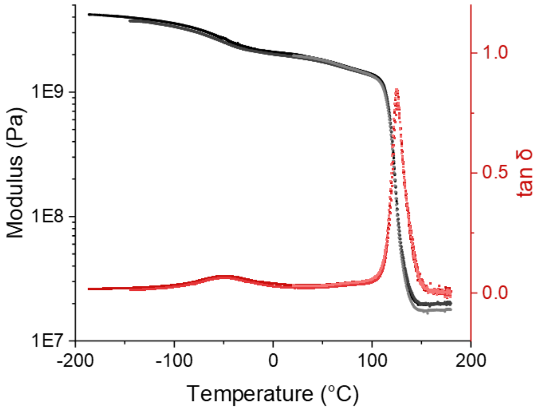

(d)

Figure SI6. Dynamic mechanical analysis for the four networks made with DER 354 and (a) MXDA, (b) PXDA, (c) 1,3-BAC & (d) 1,4-BAC

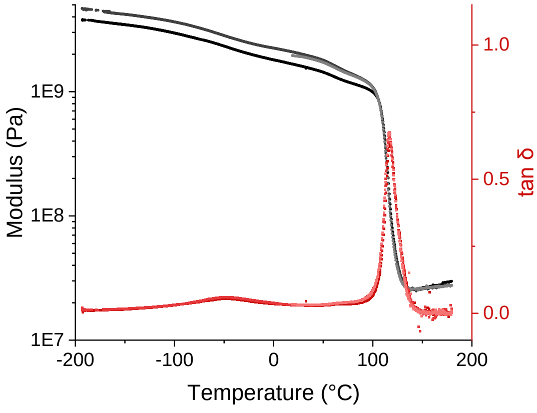

Figure SI7. Dynamic mechanical analysis for the network made with ppDGEBF and MXDA.

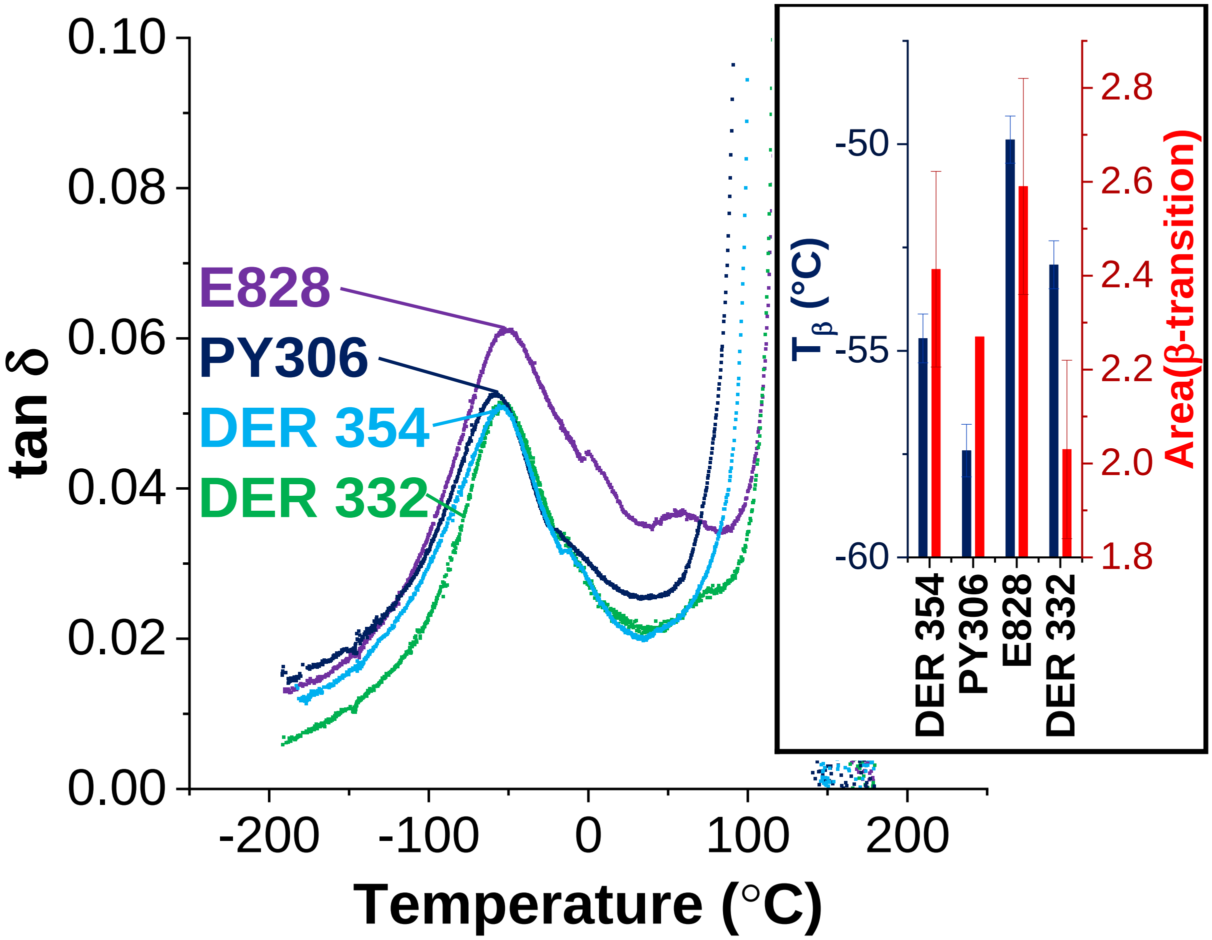

Figure SI8. tan δ against temperature for networks prepared with varied epoxy monomer and MXDA, showing the response for the β-transition. Inset: The T_β and area of the β-transition for those networks. Error bars show standard error of two samples.

(note: the comparable amine graph is shown in the manuscript – Figure 12)

Solvent sorption/desorption

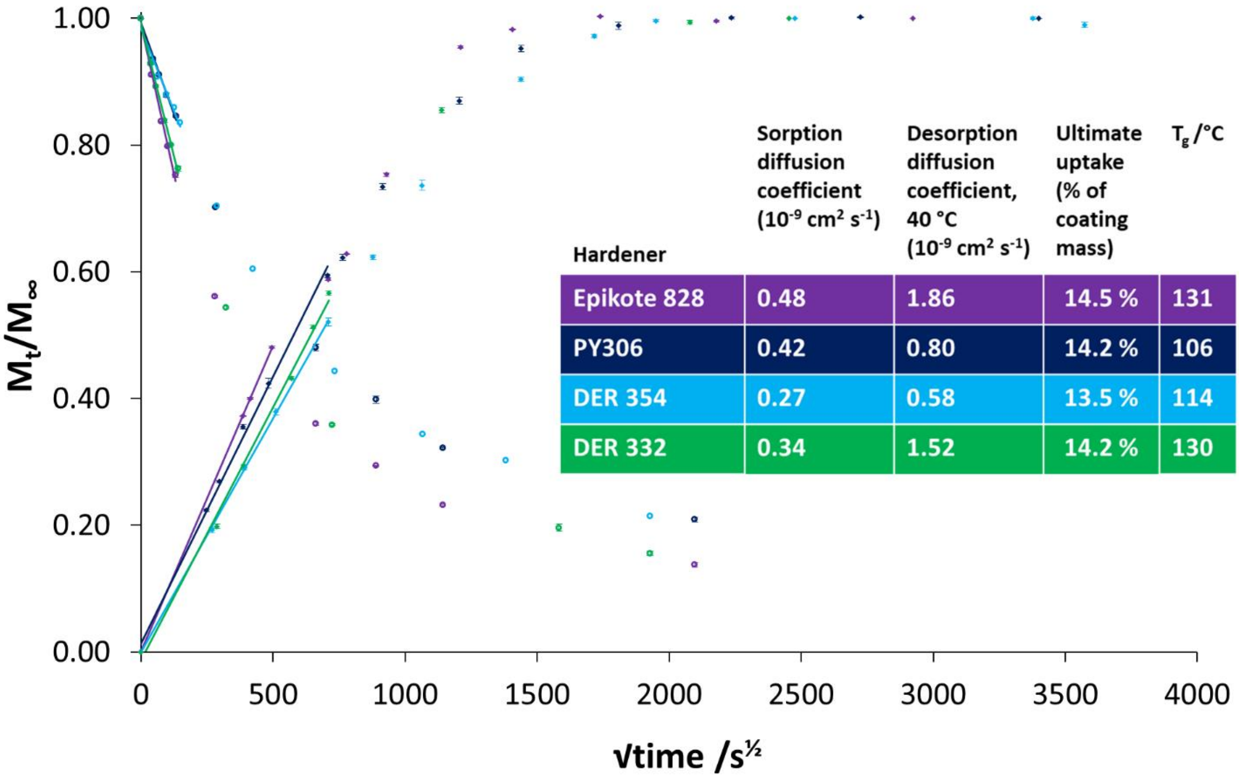

Hardener	Sorption diffusion coefficient (10^{-9} cm² s⁻¹)	Desorption diffusion coefficient, 40 °C (10^{-9} cm² s⁻¹)	Ultimate uptake (% of coating mass)	T_g /°C
Epikote 828	0.48	1.86	14.5 %	131
PY306	0.42	0.80	14.2 %	106
DER 354	0.27	0.58	13.5 %	114
DER 332	0.34	1.52	14.2 %	130

Figure SI9. Methanol ingress (beginning at the origin) and egress (beginning at y = 1) for the four networks made with MXDA and DER 354, PY306, E828 & DER 332. Lines of best fit for the initial linear portion of the data used for the calculation of diffusion coefficients are also shown.

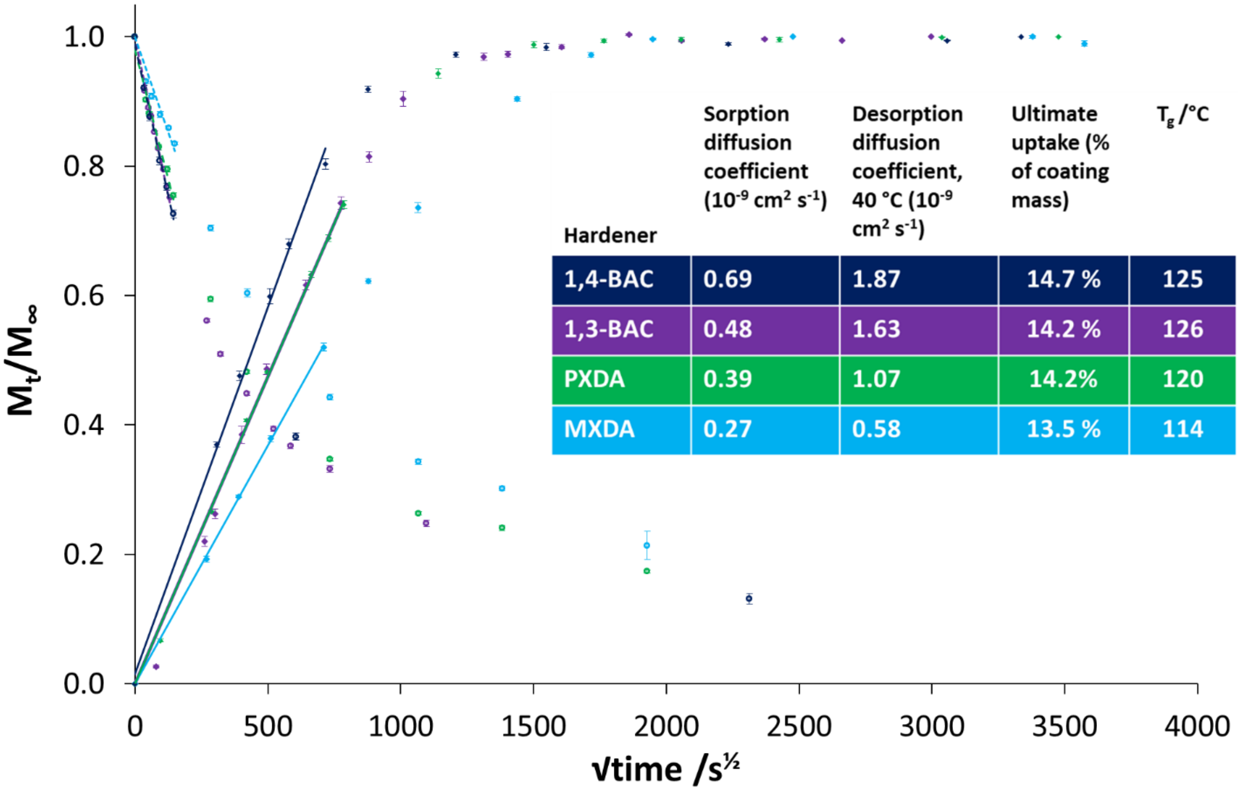

Hardener	Sorption diffusion coefficient (10^{-9} cm² s⁻¹)	Desorption diffusion coefficient, 40 °C (10^{-9} cm² s⁻¹)	Ultimate uptake (% of coating mass)	T_g /°C
1,4-BAC	0.69	1.87	14.7 %	125
1,3-BAC	0.48	1.63	14.2 %	126
PXDA	0.39	1.07	14.2%	120
MXDA	0.27	0.58	13.5 %	114

Figure SI10. Methanol ingress (beginning at the origin) and egress (beginning at y = 1) for the four networks made with DER 354 and MXDA, PXDA, 1,3-BAC & 1,4-BAC. Lines of best fit for the initial linear portion of the data used for the calculation of diffusion coefficients are also shown.

Summary table

Table SI4. Summary table for all networks in this work. Errors for T_g, modulus, crosslink density and ultimate uptake shown are standard error from three samples. Errors relating to the β-transition shown are standard error from two samples. Errors relating to $D_{sor/des}$ are based upon the standard error for the straight line fit in the initial stage of uptake, from data points which are an average of three repeats. For degree of cure and density, the significant figure for the limit of instrument resolution is shown.

Epoxy	Amine	Degree of cure (%) [NIR]	T_g (°C) [DMA]	T_β (°C) [DMA]	β-trsn peak area [DMA]	Modulus at T_g + 40 K (MPa) [DMA]	Crosslink Density (mol m^{-3}) [DMA]	Density (g cm^{-3}) [Pycnometry]	Ultimate uptake (MeOH) (% by weight)	D_{sor} (MeOH) (10^{-9} cm^2 s^{-1})	D_{des} (MeOH) (10^{-9} cm^2 s^{-1})
DER 354	MXDA	100.(0)	113.3 ± 0.2	-54.7 ± 0.6	2.4 ± 0.2	17.4 ± 2.2	1631 ± 118	1.216(9)	13.5 ± 0.09	0.26 ± 0.005	0.58 ± 0.06
PY306	MXDA	99.(8)	106.9 ± 0.2	-57.4 ± 0.6	2.3 ± 0.1	14.9 ± 1.3	1421 ± 72	1.216(0)	14.2 ± 0.07	0.42 ± 0.02	0.80 ± 0.04
E828	MXDA	100.(0)	131.9 ± 0.3	-49.9 ± 0.6	2.6 ± 0.2	23.4 ± 2.9	2111 ± 150	1.184(6)	14.5 ± 0.04	0.48 ± 0.001	1.86 ± 0.09
DER 332	MXDA	100.(0)	129.7 ± 0.2	-52.9 ± 0.6	2.0 ± 0.2	22.8 ± 2.4	2062 ± 123	1.185(6)	14.2 ± 0.07	0.34 ± 0.01	1.51 ± 0.05
ppDGEBF	MXDA	100.(0)	116.9 ± 0.2	-47.4 ± 0.1	2.5 ± 0.2	26.5 ± 0.5	2471 ± 25	1.221(0)	13.0 ± 0.03	0.22 ± 0.001	0.92 ± 0.04
DER 354	PXDA	99.(6)	120.6 ± 0.1	-53.7 ± 0.6	2.9 ± 0.1	19.1 ± 1.7	1767 ± 89	1.216(7)	14.2 ± 0.03	0.39 ± 0.005	1.07 ± 0.09
DER 354	1,3-BAC	100.(0)	127.3 ± 0.2	-55.0 ± 0.6	2.6 ± 0.1	17.8 ± 0.7	1621 ± 36	1.192(3)	14.2 ± 0.1	0.48 ± 0.01	1.63 ± 0.05
DER 354	1,4-BAC	99.(8)	124.9 ± 0.0	-49.0 ± 0.6	3.0 ± 0.1	19.1 ± 1.2	1750 ± 63	1.191(7)	14.7 ± 0.06	0.69 ± 0.02	1.87 ± 0.08

References

(1) Knox, S. T.; Wright, A.; Cameron, C.; Patrick Anthony Fairclough, J. Well-Defined Networks from DGEBF—The Importance of Regioisomerism in Epoxy Resin Networks. *Macromolecules* **2019**, *52* (18), 6861–6867. https://doi.org/10.1021/acs.macromol.9b01441.

(2) Jackson, M.; Kaushik, M.; Nazarenko, S.; Ward, S.; Maskell, R.; Wiggins, J. Effect of Free Volume Hole-Size on Fluid Ingress of Glassy Epoxy Networks. *Polymer* **2011**, *52* (20), 4528–4535. https://doi.org/10.1016/j.polymer.2011.07.042.

(3) Sahagun, C. M.; Knauer, K. M.; Morgan, S. E. Molecular Network Development and Evolution of Nanoscale Morphology in an Epoxy-Amine Thermoset Polymer. *J. Appl. Polym. Sci.* **2012**, *126* (4), 1394–1405.

(4) Poisson, N.; Lachenal, G.; Sautereau, H. Near- and Mid-Infrared Spectroscopy Studies of an Epoxy Reactive System. *Vib. Spectrosc.* **1996**, *12* (2), 237–247. https://doi.org/10.1016/0924-2031(96)00027-6.
